# Contribution of Serum Lipid Profiles to Outcome After Endovascular Thrombectomy for Anterior Circulation Ischemic Stroke

**DOI:** 10.1007/s12035-018-1391-3

**Published:** 2018-10-23

**Authors:** Slaven Pikija, Laszlo K. Sztriha, Monika Killer-Oberpfalzer, Friedrich Weymayr, Constantin Hecker, Christian Ramesmayer, Larissa Hauer, Johann Sellner

**Affiliations:** 1 Department of Neurology, Christian Doppler Medical Center, Paracelsus Medical University, Ignaz-Harrer-Straße 79, 5020 Salzburg, Austria; 2 Department of Neurology, King’s College Hospital, Denmark Hill, London, UK; 3 Research Institute for Neurointervention, Christian Doppler Medical Center, Paracelsus Medical University, Salzburg, Austria; 4 Division of Neuroradiology, Christian Doppler Medical Center, Paracelsus Medical University, Salzburg, Austria; 5 Department of Psychiatry, Psychotherapy and Psychosomatics, Christian Doppler Medical Center, Paracelsus Medical University, Salzburg, Austria; 6 Department of Neurology, Klinikum rechts der Isar, Technische Universität München, Munich, Germany

**Keywords:** Ischemic stroke, Cholesterol, Low-density lipoprotein, Thrombectomy, Outcome

## Abstract

The contribution of lipids, including low- and high-density lipoprotein cholesterol (LDL-C and HDL-C, respectively) and triglycerides (TG), to stroke outcomes is still debated. We sought to determine the impact of LDL-C concentrations on the outcome of patients with ischemic stroke in the anterior circulation who received treatment with endovascular thrombectomy (EVT). We performed a retrospective analysis of consecutive patients with acute ischemic stroke treated at a tertiary center between 2012 and 2016. Patients treated with EVT for large artery occlusion in the anterior circulation were selected. The primary endpoint was functional outcome at 3 months as measured with the modified Rankin Scale (mRS). Secondary outcome measures included hospital death and final infarct volume (FIV). Blood lipid levels were determined in a fasting state, 1 day after admission. We studied a total of 174 patients (44.8% men) with a median age of 74 years (interquartile range [IQR] 61–82) and median National Institutes of Health Stroke Scale at admission of 18 (14–22). Bridging therapy with intravenous tissue-plasminogen activator (t-PA) was administered in 122 (70.5%). The median LDL-C was 90 mg/dl (72–115). LDL-C demonstrated a U-type relationship with FIV (*p* = 0.036). Eighty-three (50.0%) patients had an mRS of 0–2 at 3 months. This favorable outcome was independently associated with younger age (OR 0.944, 95% CI 0.90–0.99, *p* = 0.012), thrombolysis in cerebral infarction 2b-3 reperfusion (OR 5.12, 95% CI 1.01–25.80, *p* = 0.015), smaller FIV (0.97 per cm^3^, 95% CI 0.97–0.99, *p* < 0.001), good leptomeningeal collaterals (OR 5.29, 95% CI 1.48–18.9, *p* = 0.011), and LDL-C more than 77 mg/dl (OR 0.179, 95% CI 0.04–0.74, *p* = 0.018). A higher LDL-C concentration early in the course of a stroke caused by large artery occlusion in the anterior circulation is independently associated with a favorable clinical outcome at 3 months. Further studies into the pathophysiological mechanisms underlying this observation are warranted.

**Electronic supplementary material:**

The online version of this article (10.1007/s12035-018-1391-3) contains supplementary material, which is available to authorized users.

## Introduction

Stroke continues to be major health burden around the world [1]. The INTERSTROKE study revealed that 10 modifiable risk factors account for 90% of strokes globally, with hypertension ranked as the most important one [2]. Further studies of modifiable factors that influence outcome are very much needed in basic science, translational, and clinical research.

Hyperlipidemia is among the major risk factors for adverse outcome in cardio- and cerebrovascular diseases. Indeed, current guidelines strongly recommend the control of total cholesterol (TC) and low-density cholesterol (LDL-C) levels in order to lower the risk of atherosclerosis and subsequent stroke [3]. Interestingly, studies trying to establish a simple linear relationship between TC, LDL-C, and stroke have revealed conflicting data. Several studies have found that lower TC in patients with acute ischemic stroke is associated with more severe disease and unfavorable outcomes [4–9]. Nevertheless, studies showing a positive effect of TC lowering on the risk for the development and progression of cardio- and cerebrovascular diseases are numerous [10]. Moreover, prior statin intake resulted in better functional outcome and decreased mortality after ischemic stroke [11–13]. In patients treated with thrombolysis for acute cerebral ischemia, independent predictors of 3-month mortality were lower HDL-C and triglyceride levels [14]. Importantly, a recent study demonstrated a U-curve relationship of TC where a lower frequency of prior ischemic stroke was associated with a high cholesterol level of ≤ 5.5 mmol/l on admission [15]. The opposite relationship was identified for patients with cholesterol > 5.5 mmol/l. Thus, cholesterol may be a factor contributing to both protective and detrimental mechanisms of action in acute ischemic stroke.

Therefore, we aimed to clarify the impact of the serum lipid profile on outcomes in acute ischemic stroke, and selected a cohort of patients undergoing endovascular therapy (EVT) for large artery occlusion in the anterior circulation.

## Subjects and Methods

We performed a retrospective review of all consecutive stroke patients admitted to Christian Doppler Medical Center, a tertiary hospital in Salzburg, Austria. The protocol was in accordance with the ethics guidance of our hospital’s Committee for the Protection of Human Subjects (Protocol UN 2553). According to Austrian regulations, informed consent is not required for the routine collection of clinical and radiological data as used in this study. A written approval for the retrospective analysis of data of patients with acute ischemic stroke was obtained from the local Ethics Committee (415-EP/73/750-2017).

The study period was 2012–2016 with inclusion criteria as follows: ≥ 18 years of age, CT angiography (CTA) or MR angiography (MRA) confirmed internal carotid artery (ICA), and/or middle cerebral artery (MCA) occlusion within 6 h of symptom onset, and EVT was performed with a stent retriever. Blood lipids (including LDL-C) were determined the day after admission in a fasting state. EVT was conducted according to the standard operating procedures of our center, implemented on the basis of available high-level evidence. Details of the scanners, imaging protocols, and methodology for determining the size of the hyperdense MCA thrombus were reported previously [16]. Extensive stroke diagnostic workup was performed in all patients, including 24-h ECG (or 72-h ECG when indicated), transthoracic, and, when needed, transesophageal cardiac ultrasound, neurovascular ultrasound of the extracranial and intracranial vessels, and autoimmune and thrombophilia panels as required. The etiology of stroke was established as per the Trial of Org 10172 in Acute Stroke (TOAST) criteria [17]. A detailed timeline of intravenous thrombolysis (IVT) and EVT was recorded as well as the outcome of the recanalization attempt using the thrombolysis in cerebral infarction (TICI) score (TICI). Follow-up CT/MRI within 24 h after EVT was used to determine the presence of intracerebral hemorrhage (ICH). Follow-up CT scans performed between 24 h and 7 days were examined to determine the final infarct volume (FIV) or any additional hemorrhagic complications. The infarct area was manually delineated on each CT slice (4 mm thickness) producing an area in square centimeters. Finally, the volume in cubic centimeters was derived from the measured area and the corresponding slice thickness [18]. Additional variables included demographic and laboratory data, leukocyte (neutrophil and lymphocyte) count on admission, National Institutes of Health Stroke Scale (NIHSS) on admission, and the modified Rankin Scale (mRS) at 3 months. The neutrophil count was divided with the leukocyte count to derive the neutrophil-to-lymphocyte ratio (NLR).

Patients were divided into groups of successful (TICI score 2b or 3) or unsuccessful (TICI score 0, 1, or 2a) recanalization.

Leptomeningeal collateral supply was assessed on pre-procedural CT angiography. We used a three-category scoring system adapted from [19]: 0—absence of collaterals in the symptomatic hemisphere, 1—less visibility of collaterals in the symptomatic hemisphere, and 2—equal or greater than the contralateral hemisphere.

We defined intracranial hemorrhage (ICH) as per the Heidelberg Bleeding Classification [20]. Patients in whom intracranial hemorrhage was clearly associated with the procedure itself (procedure-related ICH), such as due to vessel perforation, were not accounted for in the ICH group.

### Statistical Analysis

Patient demographics were summarized using descriptive statistics. Depending on the normality of distribution (as tested by the Kolmogorov-Smirnov test), continuous variables were compared using the *t* test for independent samples, or the Mann-Whitney *U* test. Categorical variables were compared using Fisher’s exact or the chi-square test, as appropriate. For the multivariate analyses, binary logistic regression was performed to calculate odds ratios. The covariates were continuous unless stated otherwise: age, NIHSS at admission, leptomeningeal vascularity (binary), thrombectomy outcome (binary), FIV in cubic centimeters, and history of statin use (binary). A *p* value of 0.05 was used as threshold for statistical significance. All statistical analyses were performed using STATA software 13.0 (StataCorp LLC, TX, USA).

## Results

Of the 204 patients receiving EVT for large artery anterior circulation stroke, we identified 174 (85.2%) who had fasting blood lipids, including LDL-C, available. The median age of these patients was 74 years (interquartile range [IQR] 61–82), 78 (44.8%) were men, and the median NIHSS score was 18 (IQR 14–22). Pre-treatment with iv t-PA was administered in 122 (70.5%). The median fasting LDL-C was 90 mg/dl (IQR 72–115). More detailed baseline characteristics are shown in Supplemental Table S1.

A total of 60 (34.7%) patients were pre-treated with a statin medication. We found that patients with a fasting LDL above normal range (> 100 mg/dl, 41.4%) were more likely to have been pre-treated with statins (58.3 vs. 32.7%, *p* = 0.002).

In all patients, a stent retriever-based procedure was applied. There were 40 (22.9%) ICA + MCA tandem occlusions. Acute stenting of an ICA occlusion was performed in 16 (32.0%) patients. Successful reperfusion, defined as TICI grade 2b or 3, was achieved in 129 (75.0%). Symptomatic hemorrhage occurred in 17 (9.8%). Good outcome (mRS 0–2) was present in 83 (50.0%) patients.

**Fig. 1:**
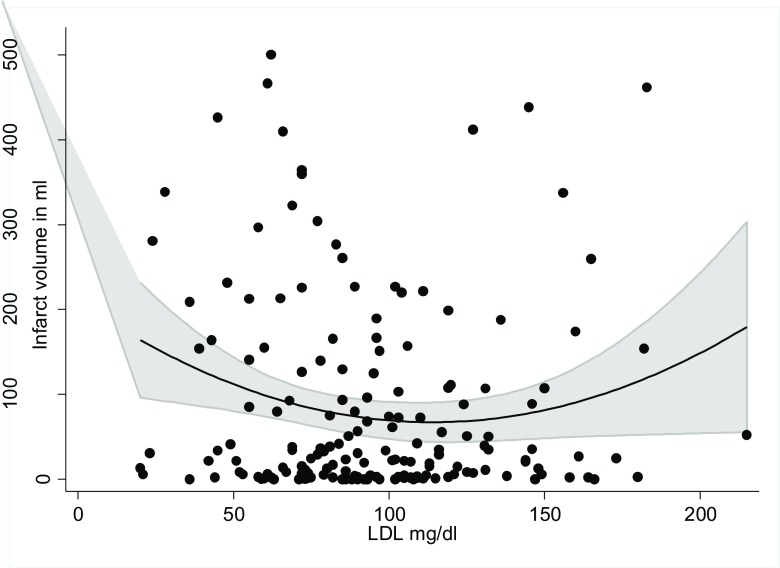
*U*-shape relationship of low-density lipoprotein with infarct volume in patients treated for large artery anterior stroke with endovascular thrombectomy

Patient demographics are shown in Table 1. LDL-C was not associated with FIV, analyzed either as a continuous variable for a linear relationship or as a binary one defined as hyperlipidemia present or absent. However, after grouping LDL-C in tertiles (1st tertile, 20–77; 2nd, 78–105; 3rd, 106–215 mg/dl), we found a *U*-shape relationship with FIV (*p* = 0.036, Fig. 1). Furthermore, LDL-C was significantly linked with good clinical outcome (mRS 0–2) in a sub-population with good reperfusion (TICI 2b, 3; *p* < 0.001), whereas there was no such relationship in cases with miserable reperfusion (TICI 0-2a; *p* = 0.104).

**Table 1:** Demographics and clinical variables of 174 patients treated with endovascular thrombectomy due to large artery anterior stroke. Range or percentages are shown in brackets

Parameter	Value
Age, median (IQR)	74 (61–82)
Male sex	78 (44.8)
Hypertension	110 (63.2)
Diabetes mellitus	20 (11.6)
Atrial fibrillation	67 (38.5)
Good outcome at 3 months (data on *n* = 166)	83 (50)
Hospital death	22 (12.8)
Admission values	
Pre-morbid mRS > 1	6 (3.4)
Body mass index	25.6 (22.8–29.0)
NIHSS	18 (14–22)
Occlusion site	
MCA M1	134 (77.0)
ICA + M1/M2	40 (22.9)
ASPECTS	8 (8–9)
Serum glucose	119 (106–138)
Creatinine	0.87 (0.77–1.07)
Total cholesterol	155 (128–185)
LDL	90 (72–115)
HDL	44 (37–55)
Triglycerides	100 (75–145)
Neutrophil to lymphocyte ratio	3.65 (2.13–5.85)
Hyperdense thrombus area	22.6 (13.6–42.6)
Stroke etiology	
Cardioembolic	141 (81.0)
Large artery atherosclerosis + other	33 (18.9)
Procedure related	
Good leptomeningeal collaterals	46 (29.3)
Intravenous thrombolysis	122 (70.5)
Time to first imaging, min	96 (66–137)
Time to needle	110 (85–150)
Time to groin puncture	189 (154–237)
Number of passes>3	67 (47.9)
Intervention time	52 (23–93)
ICA Stenting	16 (32.0)
TICI 2b or 3	129 (75.0)
Symptomatic hemorrhage	17 (9.8)
Final infarct volume	30.9 (4.3–126.5)

*IQR* interquartile range, *mRS* modified Rankin Scale, *ASPECTS* Alberta Stroke Program Early CT Score, *LDL* low-density lipoprotein, *HDL* high-density lipoprotein, *TICI* thrombolysis in cerebral infarction, *MCA* middle cerebral artery, *ICA* internal cerebral artery

**Fig. 2:**
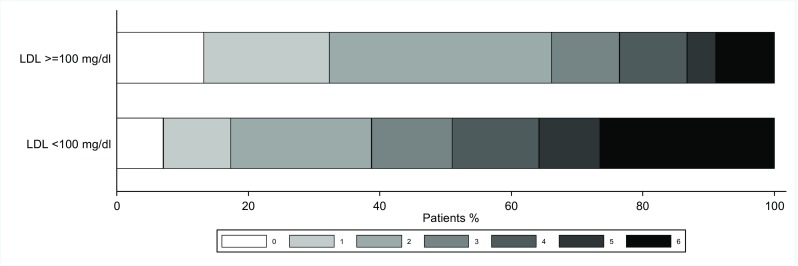
mRS outcomes at 3 months according to low or high LDL-C levels in 174 patients treated with endovascular thrombectomy for large artery anterior circulation stroke

We did not find an association between fasting LDL-C and NIHSS on admission (*p* = 0.072). LDL-C demonstrated a positive linear correlation with BMI (*p* = 0.001), and a negative such relationship with age (*p* < 0.001). The same was true for TG and age (*p* < 0.001), but not HDL-C where no relationship with age was found. Male gender was associated with higher LDL-C and HDL-C (median LDL-C 101 mg/dl [IQR 77–124] vs. 86 mg/dl [IQR 69–107] [*p* = 0.023] and median HDL-C 41 mg/dl [IQR 32–47] vs. 50 mg/dl [IQR 40–59] [*p* < 0.001] for men and women, respectively). There was no gender difference for TG. Gender did not impact on the outcome. Figure 2 demonstrates clinical outcome as measured by grades of mRS between groups of patients with LDL < 100 mg/dl vs. ≥ 100 mg/dl. Further associations between baseline characteristics, previous statin use, and LDL-C (dichotomized as < 100 mg/dl and ≥ 100 mg/dl) are described in Supplemental Tables S2 and S3. There is a significantly greater proportion of good outcome in those with prior statin use (*p* < 0.049) and LDL > 100 mg/dl (*p* < 0.001).

A multivariate logistic regression analysis showed an independent association of good outcome (mRS 0–2) with younger age (OR 0.944, 95% CI 0.90–0.99, *p* = 0.012), TICI 2b-3 reperfusion (OR 5.12, 95% CI 1.01–25.80, *p* = 0.015), smaller final infarct volume (0.97 per cm^3^, 95% CI 0.97–0.99, *p* < 0.001), good leptomeningeal collaterals (OR 5.29, 95% CI 1.48–18.9, *p* = 0.011), and LDL-C more than 77 mg/dl (OR 0.179, 95% CI 0.04–0.74, *p* = 0.018) (Table 2).

**Table 2:** Multivariate analysis of determinants for good clinical outcome (mRS 0–2)

Good outcome, mRS = 0–2			
	Odds ratio	95% Confidence interval	*p* value
Age, per year	0.944	0.902–0.987	0.012
NIHSS, per point	0.941	0.855–1.035	0.211
TICI 2b-3 reperfusion	5.119	1.015–25.801	0.015
LDL 20–77 mg/dl vs. ref.	0.179	0.043–0.742	0.018
LDL 78–105 mg/dl (ref.)			
LDL 106–215 mg/dl vs. ref.	1.593	0.496–5.109	0.434
Glucose 79–109 mg/dl vs. ref.	3.939	0.943–16.377	0.060
Glucose 110–130 mg/dl (ref.)			
Glucose 110–130 mg/dl vs. ref.	0.911	0.281–2.952	0.878
Final infarct volume in cm^3^	0.976	0.966–0.987	< 0.001
Good leptomeningeal collaterals	5.296	1.476–18.998	0.011

*vs.* versus, *Ref.* reference

When the same covariates were tested for FIV under 151 cm^3^ (i.e., 75th percentile), the total and 2nd tertile of LDL concentrations were independently associated with lower FIV, OR 3.17 (95% CI 1.17–8.54, *p* = 0.022), reflecting a *U*-relationship with FIV.

## Discussion

In this single-center study of acute ischemic stroke treated with EVT for large artery occlusion in the anterior circulation, high serum levels of LDL-C were independently associated with a better clinical outcome at 3 months. Furthermore, LDL-C showed a *U*-shape relationship with FIV, which provides a possible explanation for the previously seen ambiguity of reports on the prognostic relevance of cholesterol in acute ischemic stroke. Several previous studies have identified an association of high cholesterol levels with better stroke outcomes. Conversely, a protective effect of pre-stroke statin use in the acute phase of stroke has also been reported (Table 3).

**Table 3:** Differences of outcome surrogates depending on fasting low-density lipoprotein levels and hyperlipidemia (defined as LDL ≥ 100 mg/dl)

	Fasting LDL (mg/dl, median[IQR])		
mRS at 3 months	0–2 (*n* = 83)	3–6 (*n* = 83)	*p* value
	105 (85–124)	78 (62–102)	< 0.001
Hospital death	No (*n* = 152)	Yes (*n* = 22)	
	93 (74–119)	69 (45–93)	0.002
	Presence of hyperlipidemia		
Outcome	Present (%)	Absent (%)	*p* value
mRS 0–2 at 3 months (n = 83)	45 (54.2)	23 (27.7)	0.001
Hospital death (*n* = 22)	5 (22.7)	17 (77.3)	0.066
Final infarct volume (cm^3^)	25.8 (4.2–102.6)	32.6 (4.4–154.1)	0.702

Interval variables between groups were compared using the non-parametric Kruskal-Wallis test and comparisons between proportions using Fisher’s exact test

Stroke risk, mainly of atherothrombotic infarction [24], has been found to be elevated in those with high TC [21–23]. The favorable effects of statins include plaque stabilization and reduction of microembolism from large artery atherosclerosis [24]. While such anti-inflammatory effects of statins are beneficial, high LDL-C of > 130 mg/dl was not found to carry an increased risk of stroke in another study; therefore, more complex interactions are presumably in place [25]. In this regard, Bringeland et al. reported a *U*-curve relationship between the relative frequency of prior ischemic stroke and cholesterol level [15]. Lower frequency of prior ischemic stroke was associated with a high cholesterol level of up to 5.5 mmol/l on admission. Cholesterol levels higher than this, in contrast, were associated with an increased frequency of stroke. Of note, the *U*-type relationship with FIV in our cohort was more pronounced in statin-naïve patients. The potential relevance of cholesterol levels to recovery was also suggested by a study by Lai et al., who found that higher total cholesterol in the acute phase of ischemic stroke was a favorable prognostic factor for long-term motor function [26]. Another U-curve relationship of TC with total stroke death was reported by Iso et al. [27], with the highest rate of total stroke death occurring in patients with TC < 160 mg/dl. Koton et al. showed that patients with TC ≤ 115 mg/dl are at risk of increased stroke severity and poorer functional outcome regardless of pre-stroke statin use [4]. They also found short- and long-term mortality rates to be increased in patients with TC ≤ 115 mg/dl. In our cohort of patients in the 3rd tertile (LDL-C > 105 mg/dl), strokes were less severe at onset; however, such a relationship was not found for TC. In a previous study, a TC level above 117 mg/dl (6.5 mmol/l) was associated with better early functional outcome at 1 month. [7]. In the Copenhagen Stroke Study, higher TC levels were associated with less severe strokes and all-cause mortality in 500 patients [6]. The effect of higher TC on improved outcome is confirmed across different stroke etiologies [7]. An analysis of 1256 exclusively atherothrombotic first-ever strokes [28] found higher dependency and recurrence rates in patients in the lowest quintile of TC after 36 months; however, no effect on mortality rates between the TC quintiles was seen. Since our population consisted of 81% cardioembolic or presumed-cardioembolic stroke etiologies, direct comparison with this study is difficult.

The effect of pre-stroke statin therapy on stroke severity at admission and FIV was not observed in our population; however, we did demonstrate better outcomes at 3 months in those taking a statin (*p* = 0.013). Therefore, we were able to corroborate results of previous studies that showed statin treatment at stroke onset to be linked with favorable outcome at 90 days [29].

Whether statin treatment and cholesterol levels are risk factors for intracerebral hemorrhage is debated. A few large prospective studies (SPARCL, HPS, Cochrane review) found an elevated risk for ICH in statin users [30, 31]. However, other studies disagreed [32, 33]. A recent large registry analysis of 345,531 patients showed a lower incidence of ICH in statin users and in patients with higher cholesterol [34].

Under normal circumstances, TC metabolism is compartmentalized in the brain with no interchange with the systemic TC pool [35]. However, in pathologic states, such as when the blood-brain-barrier is disrupted by ischemia, TC could enhance repair and remyelination of penumbral tissue. Indeed, in our population, patients with good reperfusion after EVT showed association of LDL-C levels with good outcome, whereas such a relationship was not seen in those with miserable reperfusion. TC could also act as a buffer for free radicals released during ischemic injury, and therefore limit the extension of infarction [36]. The beneficial role of cholesterol for limiting brain injury is further supported by a recent study which reported increased hemorrhagic transformation in ischemic stroke patients who had low LDL levels [28].

Some shortcomings of our study include its retrospective design, lack of LDL-C measurement in 15% of our patients, and lack of information on other potential factors that could be present in patients with low LDL-C, such as decreased factor VII and albumin levels [37]. However, we did control for diabetes which also is associated with low LDL-C. We also controlled for heart failure, renal function, and body mass index. Demographics, initial stroke severity, and hospital death in our study are comparable with the findings of recently published large EVT trials. For example, NIHSS at presentation in our study (18 [IQR 14–22]) was similar to a recently published meta-analysis (NIHSS 17 [IQR 14–20]) [38]. Favorable outcome, as indicated by an mRS score of 0–2 at 3 months, was achieved in 50.0% of our patients, similar to the 46.0% reported in major trials. A mortality of 12.8% in our population also compares favorably with published data (15.3%) by Goyal et al.

In conclusion, in patients with ischemic stroke due to occlusion of a large artery in the anterior circulation of mainly cardioembolic etiology, LDL-C independently predicts outcome at 3 months, and has a *U*-curve relationship with final infarct volume. Further studies are needed to investigate the role of LDL-C in acute ischemic stroke.

## Electronic supplementary material

ESM 1 (DOCX 16.4 kb)
