# Supplementary material for: Contribution of Serum Lipid Profiles to Outcome After Endovascular Thrombectomy for Anterior Circulation Ischemic Stroke
Source: Mol Neurobiol. 2018 Oct 23;56(6):4582–8. doi: 10.1007/s12035-018-1391-3 (PMC6505499; doi:10.1007/s12035-018-1391-3)
Supplement: Supplementary file 1 — (DOCX 16.4 kb) [file 12035_2018_1391_MOESM1_ESM.docx]

Supplement

**Table S1.** Demographic characteristic of patients with and without statin use prior to treatment.

| Parameter |  | Prior statin (N=60) | No statin  (N=113) | p-value |
| --- | --- | --- | --- | --- |
| Age, median (IQR) |  | 68 (59-76) | 76 (63-84) | 0.018 |
| Male sex |  | 36 (60.0) | 42 (37.2) | 0.006 |
| Hypertension |  | 39 (65.0) | 70 (61.9) | 0.743 |
| Diabetes melitus |  | 8 (13.6) | 12 (10.6) | 0.620 |
| Atrial fibrillation |  | 18 (30.0) | 48 (42.5) | 0.139 |
| Good outcome at 3 months (n=166) |  | 35 (61.4) | 48 (44.4) | 0.049 |
| Hospital death |  | 2 (3.4) | 20 (17.9) | 0.007 |
| Admission values |  |  |  |  |
| Premorbid mRS>1 |  | 1 (1.7) | 5 (4.4) | 0.666 |
| Body mass index |  | 27.2 (24.5-29.2) | 24.9 8 (22.3-28.8) | 0.044 |
| NIHSS |  | 17 (11-22) | 18 (14-22) | 0.375 |
| Serum glucosae |  | 123 (112-145) | 117 (106-133) | 0.067 |
| Creatinine |  | 0.89 (0.77-1.01) | 0.87 (0.75-1.11) | 0.916 |
| Cholesterol |  | 177 (137-205) | 146 (127-166) | 0.001 |
| LDL |  | 114 (80-145) | 85 (69-104) | <0.001 |
| HDL |  | 41 (33-50) | 45 (38-58) | 0.014 |
| Triglycerides |  | 117 (83-155) | 94 (71-132) | 0.015 |
| Neutrophil to lymphocyte ratio |  | 3.69 (1.93-5.78) | 3.65 (2.21-6.00) | 0.747 |
| Hyperdense thrombus area (N=95) |  | 33.5 (15.8-50.2) | 20.2 (13.6-38.1) | 0.097 |
| Stroke etiology |  |  |  | 0.014 |
| Cardioembolic and unknown |  | 42 (70.0) | 98 (86.7) |  |
| Large artery atherosclerosis + other |  | 18 (30.0) | 15 (13.3) |  |

**Table S2.** Demographics and clinical variables of patients with LDL-C < and > 100 mg/dl

| Parameter |  | LDL-C <100 mg/dl (N=102) | LDL-C ≥ 100 mg/dl (N=72) | p-value |
| --- | --- | --- | --- | --- |
| Age, median (IQR) |  | 76 (70-84) | 66 (56-75) | 0.018 |
| Male sex |  | 38 (37.2) | 40 (55.6) | 0.020 |
| Hypertension |  | 63 (61.8) | 47 (65.3) | 0.750 |
| Diabetes mellitus |  | 16 (15.7) | 4 (5.6) | 0.053 |
| Atrial fibrillation |  | 46 (45.1) | 21 (29.2) | 0.040 |
| Good outcome at 3 months (N=166) |  | 38 (38.8) | 45 (66.2) | 0.001 |
| Hospital death |  | 17 (16.8) | 5 (7.0) | 0.066 |
| Admission values |  |  |  |  |
| Premorbid mRS>1 |  | 5 (4.9) | 1 (1.4) | 0.403 |
| Body mass index |  | 24.7 (22.6-27.8) | 27.0 (24.4-29.4) | 0.044 |
| NIHSS |  | 18 (15-22) | 17 (11-20) | 0.375 |
| Serum glucose |  | 121 (107-145) | 117 (201-131) | 0.066 |
| Creatinine |  | 0.87 (0.79-1.09) | 0.87(0.74-1.03) | 0.915 |
| Cholesterol |  | 134 (120-149) | 189 (168-212) | 0.001 |
| Prior statin use |  | 25 (24.8) | 35 (48.6) | 0.002 |
| HDL |  | 43 (34-55) | 44 (38-56) | 0.014 |
| Triglycerides |  | 91 (70-119) | 118 (87-163) | 0.015 |
| Neutrophil to lymphocyte ratio |  | 3.80 (2.446.00) | 3.19 (1.81-5.71) | 0.747 |
| Hyperdense thrombus area (N=95) |  | 22.3 (15.3-46.5) | 23.3 (12.1-40.1) | 0.097 |
| Stroke etiology |  |  |  | 0.116 |
| Cardioembolic |  | 87 (85.3) | 54 (75.0) |  |
| Large artery atherosclerosis + other |  | 15 (14.7) | 18 (25.0) |  |
